# Diazoxide Protects against Myocardial Ischemia/Reperfusion Injury by Moderating ERS via Regulation of the miR-10a/IRE1 Pathway

**DOI:** 10.1155/2020/4957238

**Published:** 2020-09-08

**Authors:** Lin Zhang, Shuang Cai, Song Cao, Jia Nie, Wenjing Zhou, Yu Zhang, Ke Li, Haiying Wang, Shouyang Yu, Tian Yu

**Affiliations:** ^1^Guizhou Key Laboratory of Anesthesia and Organ Protection, Affiliated Hospital of Zunyi Medical University, 563000 Zunyi, China; ^2^Key Laboratory of Brain Science, Zunyi Medical University, 563000 Zunyi, China; ^3^Department of Anesthesiology, Affiliated Hospital of Zunyi Medical University, 563000 Zunyi, China; ^4^Department of Anesthesiology, Hospital of Stomatology, Zunyi Medical University, 563000 Zunyi, China

## Abstract

Nowadays, reperfusion is still the most effective treatment for ischemic heart disease. However, cardiac reperfusion therapy would lead to reperfusion injury, which may have resulted from endoplasmic reticulum stress (ERS) during reperfusion. Diazoxide (DZ) is a highly selective mitochondrial adenosine triphosphate-sensitive potassium channel opener. Its protective effect on I/R injury has been confirmed in many organs such as the heart and brain. However, the mechanism of its protective effect has not been fully elucidated. MicroRNAs (miRNAs) are widely involved in pathologies of heart disease. In this study, we found that miR-10a expression was highly upregulated in the myocardial I/R groups, and DZ treatment significantly reduced the expression of miR-10a. More importantly, we found that DZ treatment can moderate ERS via regulation of the miR-10a/IRE1 pathway in the I/R and H/R models, thereby protecting myocardial H/R injury.

## 1. Introduction

The timely opening of infarct-related arteries and the recovery of blood supply to the ischemic myocardium are key to saving the dying cardiac muscle [1]. However, studies have found that recovery of blood perfusion after a period of ischemia in the myocardium would lead to increased damage to myocardial structure and function and decreased cardiac function and malignant arrhythmia, resulting in myocardial ischemia/reperfusion (I/R) injury [2]. At present, I/R injury is still a major problem in the treatment of cardiac ischemia. So, it is of great clinical significance to study how to reduce or even eliminate the occurrence of I/R injury on the basis of early recovery of coronary blood flow.

Diazoxide (DZ) has been demonstrated to inhibit apoptosis to limit myocardial or cerebral I/R injury. DZ is a kind of K^+^ channel agonist, and the mitochondrial ATP-dependent K^+^ (mKATP) channels have been suggested to mediate neuroprotective effects [3]. In the rat and mouse models of I/R injury, DZ preconditioning reduced the volume of brain infarcts induced by middle cerebral artery occlusion [4, 5]. On a subcellular level, the stabilization of the mitochondrial membrane potential and the preservation of mitochondrial integrity may contribute to the antiapoptotic actions of DZ in neurons [6–8]. On a molecular level, the protective effects of DZ may be mediated by the inhibition of caspase-3 activation [9, 10]. Although the protective effects of DZ have been demonstrated previously, the detailed cellular mechanisms underlying its actions against I/R-induced myocardial injury remain to be elucidated.

MicroRNAs (miRNAs) are endogenous, noncoding, single-stranded small molecules that are composed of 22-23 bases and function to regulate gene expression posttranscriptionally, [11, 12]. More and more studies have found that miRNA expression is tissue-specific. miR-21, miR-1, and miR-296 are specifically expressed in cardiomyocytes, which are involved in pathological and physiological processes such as cardiac development, myocardial apoptosis, myocardial remodeling, and heart failure [13, 14]. In recent years, the regulatory effect of miRNAs in myocardial I/R injury has received increasing attention. This study analyzed a subset of miRNAs that were differentially expressed during and after myocardial I/R injury, with miR-10a being the most variable. The miR-10 family is involved in cell growth and development. In neuroblastoma cells [15] or smooth muscle cells [16], overexpression of miR-10a/10b reduces cell proliferation and promotes differentiation. Overexpression of miR-10 in pancreatic cancer and malignant breast cancer promotes tumor invasion and metastasis [17, 18]. In addition, overexpression of miR-10b also inhibits angiogenesis by inhibiting the expression of BDNF [19]. miR-10a/10b promotes vascular growth by regulating the behavior of sophisticated cells [20]. However, whether miR-10a participates in the development of myocardial I/R injury and its mechanism of action is not clearly reported.

The endoplasmic reticulum (ER) is one of the most important organelles in eukaryotic cells regulating protein folding, Ca^2+^ homeostasis, and stress response, which is very sensitive to stress stimuli [21]. Stress factors including ischemia/hypoxia, disulfide bond formation disorders, and protein transport abnormalities can lead to dysfunction of the endoplasmic reticulum, which is endoplasmic reticulum stress (ERS) [22]. When ERS occurs, the ER protein cannot be correctly folded and thus retained in large quantities, stimulating the signaling between the ER, the Golgi, and the nucleus, leading to inhibition of protein synthesis and initiation of transcription of related genes. ERS induces upregulation of ER chaperones such as glucose regulatory proteins (GRPs), folding enzymes, and calreticulin, enhancing the ability of the ER to treat unfolded proteins and promoting the recovery of ER function [23]. When ERS persists or is at a high level, it will induce the activation of proapoptotic factors, trigger related apoptosis pathways, and induce cell apoptosis. ERS is closely related to the occurrence and development of I/R injury [24].

In this study, a rat model of myocardial I/R injury was established to observe the changes of miRNA in myocardial I/R injury. DZ treatment was performed before reperfusion to verify the protective effect of DZ on myocardial I/R injury. The effect of ERS on the development of myocardial I/R injury was discussed by further analysis of miR-10a with significant differences in changes. Furthermore, the possible molecular mechanism of miR-10a regulating ERS was explored, which provided a theoretical basis for clinical treatment of the myocardial I/R injury.

## 2. Materials and Methods

### 2.1. Animals

Sprague Dawley rats (20 ± 3 D, male and female unlimited, 60 ± 5 g) were obtained from the animal center of Third Military Medical University. All rats were kept on a 12 h light-dark cycle individually ventilated cage in 75°F with free access to food and water before the experiment. All rats were treated according to the Guide for the Care and Use of Laboratory Animals published by the US National Institutes of Health (NAP, 8th edition, Dec. 2010), and the study was approved by the Institutional Laboratory Animal Care and Use Committee of Zunyi Medical University.

### 2.2. Construction of Myocardial I/R Models

The myocardial I/R models were performed as described previously [25]. Briefly, sodium pentobarbital (45 mg/kg) was used to anesthetize rats firstly; then, thoracotomy was performed. The aorta was removed, and the heart was placed in 4°C KH buffer, cannulated through the aorta, connected to the Langendorff system, and perfused at 37°C via the aorta by 95% O_2_ and 5% CO_2_ gas fully saturated KH solution. Rats were randomly divided into 3 groups. The control group was aerobically perfused with KH solution for 120 min. The heart of the I/R group rats was hypoxia-perfused for 30 min and reperfused for 60 min after 30 min of aerobic equilibration perfusion. The DZ group was treated the same as the I/R group but perfused with 50 *μ*m DZ for 5 min before reperfusion. The DZ dissolved in DMSO solution.

### 2.3. TTC Staining

Heart samples were collected after the rats were sacrificed and placed into a refrigerator (-80) for 8 min, then cut into slices with a thickness of about 2~3 mm each. Slices were immersed for 30 min into 2% triphenyltetrazolium chloride (TTC) solution at 37°C in the dark. Stained slices were fixed in 4% paraformaldehyde for 30 min. Images were taken by the FSX100 microscope (Olympus, Shenzhen, China), and sizes of different stained areas were measured with the image analysis software Image-Pro Plus 6.0. The ratios of the infarct area to the left ventricular area were calculated.

### 2.4. Histological Examination

Myocardial samples were collected after the rats were sacrificed. After 4% paraformaldehyde-fixed, the heart samples were dehydrated and embedded in paraffin; hematoxylin-eosin (HE) staining was conducted. Images of stained sections were obtained by the FSX100 microscope (Olympus, Shenzhen, China).

### 2.5. miRNA Profiling

The aberrant miRNA expressions of the myocardial samples were analyzed by miRNA sequencing as previously described [26, 27] Briefly, total RNA of the tissues was extracted to prepare the small RNA sequencing library by using the NEBNext Multiplex Small RNA Library Prep Set for Illumina (NEB, USA) according to the manufacturer's instruction. The libraries were finally sequenced, and the Solexa CHASTITY quantity filtered reads were harvested as clean reads. For data analysis, differentially expressed miRNA profiles between two groups were compared, and fold change and *p* value were calculated and used to identify significant differentially expressed miRNAs, and hierarchical clustering was performed. The selected miRNAs verified the change of the expression by qRT-PCR.

### 2.6. Isolation and Purification of Rat Primary Cardiomyocytes

After being sacrificed, the rats were sterilized with 75% ethanol; the chest skin was cut and disinfected once. The surgical instrument was then replaced, and the heart was extracted and placed in a large dish containing PBS. After removing the large blood vessels attached to the surface of the heart and the atria, tissues were mixed with 5 ml collagenase and 2.5 ml 0.05% trypsin, digested at 37°C for 10 min. The supernatant was collected and resuspended in DMEM containing 10% fetal bovine serum. Cells were placed in the incubator for 2 to 3 hours until the fibroblasts were attached. The supernatant was collected and resuspended in DMEM containing 10% calf serum and BrdU (10 mM) (1 : 80), and cardiomyocytes were obtained. Obtained cardiomyocytes were identified by immunofluorescence. Cardiomyocytes were cultured in a humidified incubator at 37°C and 5% CO_2_.

### 2.7. Cardiomyocyte Hypoxia/Reoxygenation (H/R) Model

Cardiomyocytes were divided into different groups. The normal group was continuously cultured in a cell incubator under normoxic conditions (95% CO_2_+5% O_2_) until the end of the experiment. The H/R group was hypoxic for 4 hours (5% CO_2_+1% O_2_+94% N_2_) and reoxygenated (95% CO2+5% O2) for 24 h. 100 *μ*mol/l DZ was added into DMEM and incubated with cardiomyocytes for 5 min at the beginning of reoxygenation in the DZ group.

### 2.8. In Vivo and In Vitro Transfection

The miR-10a mimic, miR-10a inhibitor, and negative control oligonucleotides were purchased from Synthgene (Nanjing, China). Lipofectamine 2000 (Invitrogen, USA) was utilized to transfect the miR-10a mimic, miR-10a inhibitor, and negative control oligonucleotides (NC) according to the manufacturer's instructions. The concentration of the miR-10a mimic, miR-10a inhibitor, and NC used for transfection was 100 nM in this study. The miR-10a and IRE1 adenovirus were designed by Obio Technology (Shanghai, China) and injected into the tail vein of the experimental rat before one week of the operation.

### 2.9. MTT Assay and LDH Analysis

3-(4,5-Dimethylthiazol-2-yl)-2,5-diphenyltetrazolium bromide (MTT) assay and lactate dehydrogenase (LDH) cytotoxicity assay (Beyotime, Nanjing, China) were used to detect cardiomyocytes proliferation and lactate dehydrogenase release in the different groups according to the manufacturer's instructions. For MTT assay, cardiomyocytes were harvested at 48 h, after which 200 *μ*l of MTT solution was added to each well. After 4 h of incubation at 37°C, the MTT solution was carefully aspirated and 200 *μ*l of DMSO was added per well. The optical density of each well at 490 nm was read with a microplate reader (Molecular Devices, USA). The cell viability was calculated as follows: [(experimental release − spontaneous release)/(maximum release − spontaneous release)] × 100. For LDH assay, the supernatants of cells were collected for the measurement of LDH release by a commercial LDH cytotoxicity assay kit (Beyotime, Nanjing, China). The absorbance at 490 nm was read using a microplate reader (BioTek Instruments, Inc.).

### 2.10. Real-Time PCR

Total RNA was extracted from cardiomyocytes and rat myocardial tissues using total RNA extraction reagent (Synthgene, Nanjing, China) according to the manufacturer's instructions. For the analysis of miRNA expression, TaqMan probes (Thermo Fisher Scientific, Shanghai, China) were used according to the manufacturer's instructions. qPCR reaction was performed for 40 cycles (95°C, 30 s; 72°C, 30 s) after an initial denaturation step (95°C, 5 min) on the CFX96 system of Bio-Rad. Quantitative measurements were determined with the 2^-*ΔΔ*CT^ method; the small U6 RNA expression was used as the internal control. For the analysis of mRNA expression, qPCR was performed using an SYBR Green qPCR Mix (Vazyme, Nanjing, China) on an Applied Biosystems 7300 sequence detection system (Applied Biosystems). GAPDH was used as the internal control. The reactions were performed in a 96-well plate at 95°C for 10 min, followed by 40 cycles of 95°C for 15 s, 56°C for 15 s, and 72°C for 30 s. PCR primers were as follows: IRE1*α* FP: 5′-TAGTCAGTTCTGCGTCCGCT-3′; IRE1*α* RP: 5′-TTCCAAAAATCCCGAGGCCG-3 ′; GRP78 FP: 5′-GAACGTCTGATTGGCGATGC-3′; GRP78 RP: 5′-TCAAAGACCGTGTTCTCGGG-3′; XBP-1 FP: 5′-CTGAGTCCGCAGCAGGTG-3′; XBP-1 RP: 5′-GTCCAGAATGCCCAACAGGA-3′; GAPDH FP: 5′-GATATTGTTGACATCAATGAC-3′; GAPDH RP: 5′-TTGATTTTGGAGGGATCTCG-3′.

### 2.11. Western Blot Analysis

Total protein was extracted using RIPA lysis buffer containing 1 mM PMSF (Synthgene, Nanjing, China) from rat myocardial tissues or transfected cardiomyocytes. The rat myocardial tissues included normal control, I/R injury, and I/R injury or DZ treatment rat myocardial tissues injected miR-10a and IRE1 adenovirus. The cardiomyocytes included normal, I/R, I/R and DZ treatment cells that transfected the NC, miR-10a mimic, and miR-10a inhibitor separately. Protein concentration was measured by the BCA protein assay kit (Thermo Fisher Scientific, Waltham, China) according to the manufacturer's instructions. The experimental procedure was carried out according to the previously published protocol [28]. The extracted protein was loaded onto a 10% Bis-Tris gel (50 *μ*g per cell) and electrophoresed for 1 h at 150 V, transferred to PVDF membranes, and blocked with 5% skimmed milk at room temperature for 2 h, followed by incubation with IRE1, XBP1, and GRP78 primary antibody, respectively (1 : 1000 dilution, Proteintech, Wuhan, China) at 4°C for overnight; then, membranes were incubated with HRP-conjugated secondary antibody (1 : 3000 dilution, Proteintech, Wuhan, China) for 2 h. ECL Western blotting substrate (Synthgene, Nanjing, China) was applied to detect PVDF membranes.

### 2.12. Pull-Down Assay

The pull-down assay was carried out according to a previously described protocol [29]. Briefly, cardiomyocytes were transfected with biotinylated miR-10a (miR-10a probe) and control probe that were purchased from Synthgene (Nanjing, China). A total of 10^7^ cells were harvested and lysed by using lysis buffer. Total RNA was pretreated with DNase I and then heated at 65°C for 5 min, followed by an instant ice bath. Then, the probes were incubated with streptavidin-coated magnetic beads (New England Biolabs, USA) at 4°C for 4 h. After incubation, beads were washed and treated with TRIzol reagent to extract RNA.

### 2.13. Luciferase Reporter Analysis

1 × 10^5^ HEK 293T cells were seeded in triplicate in 6-well plates and allowed to settle for 24 h. Wild-type/mutant luciferase plasmid pGL3-IRE1-3′UTR, control luciferase plasmid, and pRL-TK Renilla plasmid were constructed by Synthgene (Nanjing, China). Plasmids were transfected into HEK 293T cells using Lipofectamine 2000 according to the manufacturer's instructions. Luciferase assays were performed 48 hours after transfection using the Dual-Luciferase Reporter Assay System (Promega, Beijing, China).

### 2.14. Immunofluorescence Analysis

For immunofluorescence staining, cardiomyocytes were blocked by incubation with 4% (*w*/*v*) BSA, incubated overnight with GRP78 primary antibody or ACTA1 primary antibody, separately. After incubation with Alexa Fluor 488 or 594 labeled secondary antibody and staining with DAPI, cardiomyocytes were observed under a confocal microscope (Leica, Shanghai, China).

### 2.15. Statistical Analysis

Results are collected from three independent experiments. All the results are presented as mean ± SD. Results were analyzed with one-way ANOVA and *t*-test using GraphPad Prism 6.0; *p* < 0.05 is considered statistic significant.

## 3. Results

### 3.1. Effects of I/R Injury and DZ Treatment on the Expression of miRNAs in Rat Myocardial Tissue

**Figure 1:**
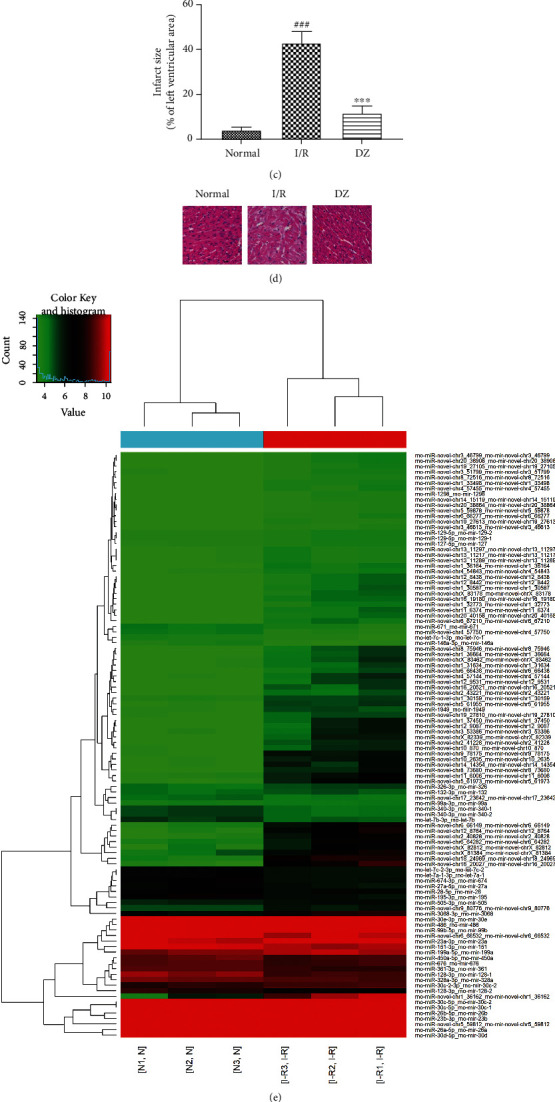
Effects of I/R injury and DZ treatment on the expression of miRNAs in rat myocardial tissue. (a) The schematic diagram of the established myocardial I/R injury rat model. (b, c) Representative images of hearts from the NC, I/R, and DZ rats, hearts were stained with TTC to reveal infarcted tissue, the red area represents the viable myocardium, and the white area represents ischemic dead tissue. Scale bar = 1 mm. (d) Rat myocardial tissues in different groups were detected by HE staining. Scale bar = 20 *μ*m. (e) RNA sequencing detects the miRNA expression difference in normal and I/R injury myocardial tissues. (f) miRNA screening: miRNA expression in the myocardium was assessed by qRT-PCR. Data were expressed as mean ± SD (*n* = 6). ^#^*p* < 0.05, ^##^*p* < 0.01, and ^###^*p* < 0.001, compared to the normal group; ^∗^*p* < 0.05, ^∗∗^*p* < 0.01, and ^∗∗∗^*p* < 0.001, compared to the I/R group.

To investigate the effects of I/R injury and DZ treatment on the expression of miRNAs in rat myocardial tissue, we established a rat model of myocardial I/R injury; the process is shown in Figure 1(a). I/R injury can induce myocardial infarction (MI), TTC staining was used as a confirmation of consistent induction of MI, the red area represents the viable myocardium, and the white area represents ischemic dead tissue. Compared with the sham group, the infarct size of the I/R group increased significantly, but DZ can protect cardiomyocytes from MI and reduced infarct size (Figures 1(b) and 1(c)). According to the results of HE staining, we found that the sham group has strict rules of myofilament and complete sarcomere morphology (Figure 1(d)). In the I/R group, the myofilament is confused and cardiomyocytes rupture, together with sarcomere contracture deformation and sarcoplasmic reticulum expansion. In the DZ group, the damage was alleviated compared to the I/R group, and the myofilament was arranged neatly. The results show that the model was successfully established. Afterward, in order to conform DZ effect I/R injury through miRNA, we used sequencing and qPCR to detect the expression of miRNAs in myocardial tissue (Figures 1(e) and 1(f)). The difference in miR-10a was most pronounced among all miRNAs tested. Compared with the sham group, the expression of miR-10a was significantly increased in the I/R group, and the expression of miR-10a was significantly inhibited after treatment with DZ. Based on these findings, diazoxide protects against myocardial ischemia/reperfusion injury, possibly by regulating miR-10a.

### 3.2. Upregulation of miR-10a Is Involved in H/R-Induced Cardiomyocyte Injury

**Figure 2:**
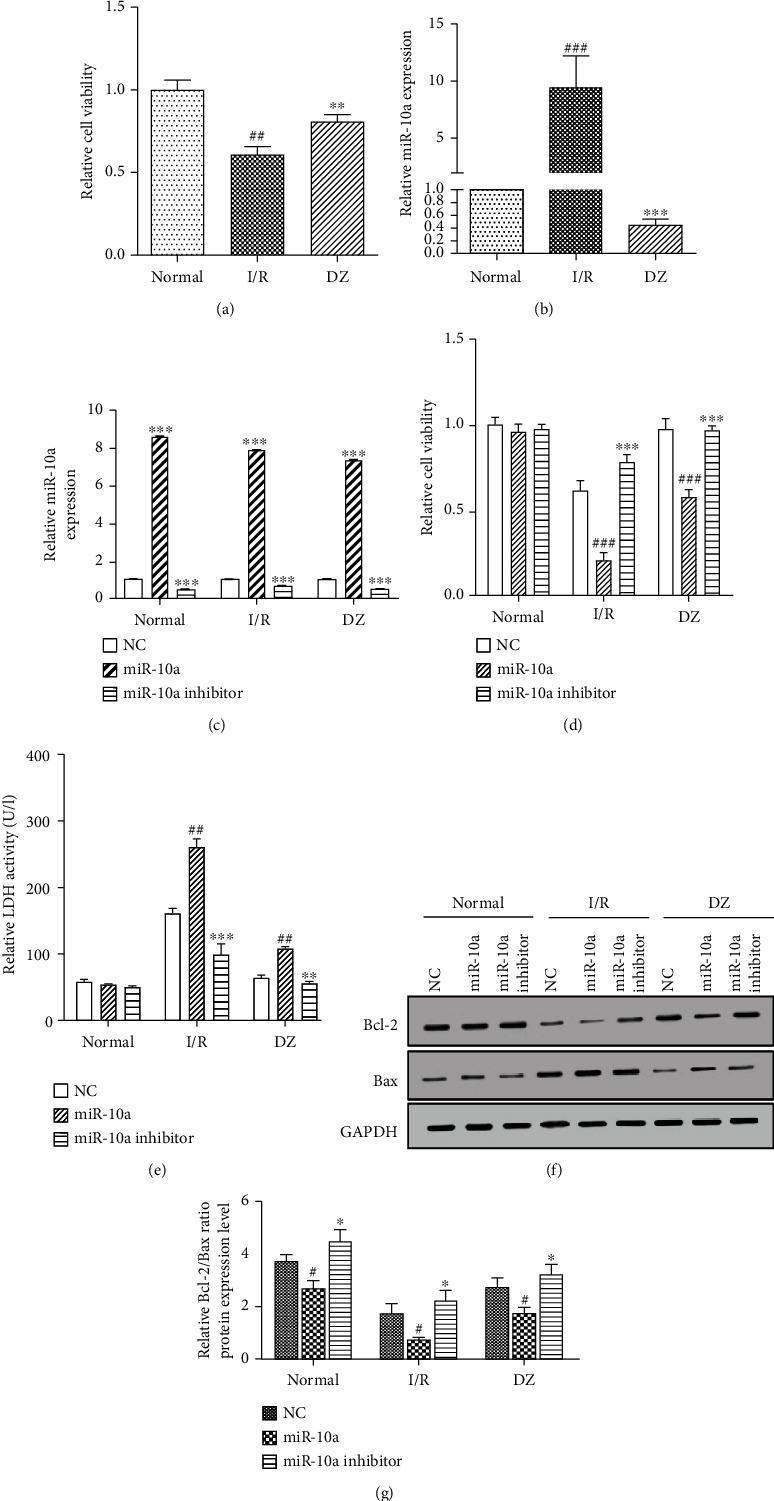
Upregulation of miR-10a is involved in H/R-induced cardiomyocyte injury. (a) MTT assay was used to detect the cell viability of each group. (b) miR-10a expression in cardiomyocytes was assessed by qRT-PCR. (c) The transfection efficiency of the miR-10a mimic and miR-10a inhibitor was detected by qRT-PCR in cardiomyocytes. (d) The effect of the miR-10a mimic and miR-10a inhibitor on cell viability of each group was detected by MTT analysis. (e) The effect of the miR-10a mimic and miR-10a inhibitor on LDH release of each group was detected by LDH analysis. (f) Protein expressions of Bcl-2 and Bax were detected using Western blot. (g) Quantitative analysis of (f). Data were expressed as mean ± SD in three independent experiments. For (a) and (b), ^##^*p* < 0.01 and ^###^*p* < 0.001, compared to the normal group; ^∗∗^*p* < 0.01 and ^∗∗∗^*p* < 0.001, compared to the I/R group. For (c), ^∗∗∗^*p* < 0.001, compared to the NC group. For (d)–(f), ^#^*p* < 0.05, ^##^*p* < 0.01, and ^###^*p* < 0.001, compared to the NC group; ^∗^*p* < 0.05, ^∗∗^*p* < 0.01, and ^∗∗∗^*p* < 0.001, compared to the miR-10a group.

I/R can significantly increase the expression of miR-10a in myocardial tissue. To further explore the role of miR-10a in myocardial injury, we established an H/R injury model using rat primary cardiomyocytes. Rat primary cardiomyocytes were isolated from rat cardiac tissue and identified by immunofluorescence. The identification results were shown in the supplementary data. The immunofluorescence results confirmed the cells were rat cardiomyocytes. As shown in Figure 2(a), the cell viability of the H/R group was significantly inhibited as compared with the control group, and the cell viability of the DZ group was increased compared with the H/R group. Next, we examined the expression of miR-10a. As a result (Figure 2(b)), the expression of miR-10a was significantly increased in the H/R group compared with the control group, and the expression of miR-10a was restored in the DZ group, which was consistent in rat myocardial tissue. To further investigate the cardiomyocyte injury of miR-10a, we transfected the miR-10a mimic and miR-10a inhibitor in cardiomyocytes and detected the transfection efficiency. The transfection efficiency results are shown in Figure 2(c). Then, we detected myocardial cell function-related indicators after treatment. MTT (Figure 2(d)) and LDH test results (Figure 2(e)) showed that, compared with the control group, H/R was able to inhibit cell viability and increase the level of LDH in the cardiomyocyte culture supernatant; DZ treatment could restore the cell viability and LDH expression level, indicating that the model was successfully constructed. In addition, the miR-10a mimic can cause cell damage and LDH can increase cardiomyocytes in the H/R group and DZ group; DZ treatment alleviates the effect of miR-10a on cell damage and LDH release. However, the H/R damage effect can be reversed by the miR-10a inhibitor. The miR-10a inhibitor can protect cardiomyocytes from H/R injury, that is, increase myocardial cell viability and reduce LDH levels in the supernatant. At the same time, we examined the protein expression levels of Bcl-2 and Bax by Western blot (Figure 2(f)) and quantitatively analyzed the Bcl-2 and Bax expression ratio (Figure 2(g)). It was found that the H/R injury caused a decrease in the expression ratio of Bcl-2 and Bax, the miR-10a mimic aggravated this phenomenon, and the miR-10a inhibitor reversed the decrease of the Bcl-2 and Bax expression ratio. DZ slows down miR-10a mimic or inhibitor effects on Bcl-2 and Bax expression in H/R group cells. In conclusion, upregulation of miR-10a is involved in H/R injury in cardiomyocytes, and diazoxide protects against myocardial ischemia/reperfusion injury by regulating miR-10a.

### 3.3. ERS Is Involved in I/R Injury

**Figure 3:**
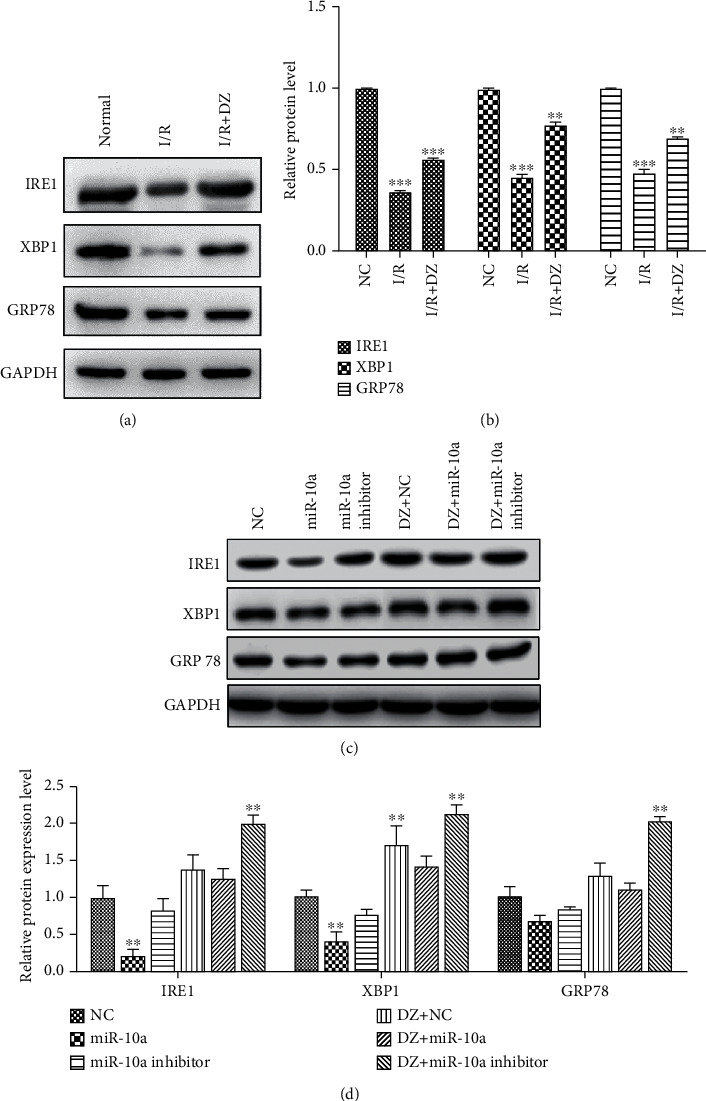
ERS is involved in I/R injury. (a) The effects of I/R and DZ treatment on protein expressions of GRP78, IRE1, and XBP1 were detected using Western blot. (b) Histogram summarizing results in (a). (c) The effects of the miR-10a mimic, miR-10a inhibitor, and DZ treatment separately or simultaneously on protein expressions of GRP78, IRE1, and XBP1 were detected using Western blot. (d) Histogram summarizing results in (c). Data were expressed as mean ± SD in three independent experiments. ^∗∗^*p* < 0.01 and ^∗∗∗^*p* < 0.001, compared to the NC group.

After successfully establishing the model of I/R in rat primary cardiomyocytes, we also examined changes in ERS-related protein expression (Figure 3(a)). Compared with the control group, the expression of ERS marker proteins GRP78, IRE1, and XBP1 in the H/R group was significantly downregulated, and DZ treatment rescued the expression of ERS marker proteins (Figure 3(b)). The results indicated that ERS is inhibited in the early stage of myocardial H/R injury. At the same time, we transfected the miR-10a mimic and miR-10a inhibitor in the H/R group and DZ group cardiomyocytes, respectively. Then, we detected the expression of ERS-related proteins (Figure 3(c)). Compared with the NC group, the miR-10a mimic significantly reduced the expression of ERS-related proteins GRP78, IRE1, and XBP1, while the miR-10a inhibitor reversed the inhibitory effect of miR-10a. DZ diminished the effect of miR-10a on ERS-related proteins expression compared with the H/R group cells (Figure 3(d)). In combination, the effect of the miR-10a mimic is similar to that of H/R injury, which can inhibit moderate ERS and cause damage to cardiomyocytes.

### 3.4. IRE1 Is the Target Gene of miR-10a in Cardiomyocytes

**Figure 4:**
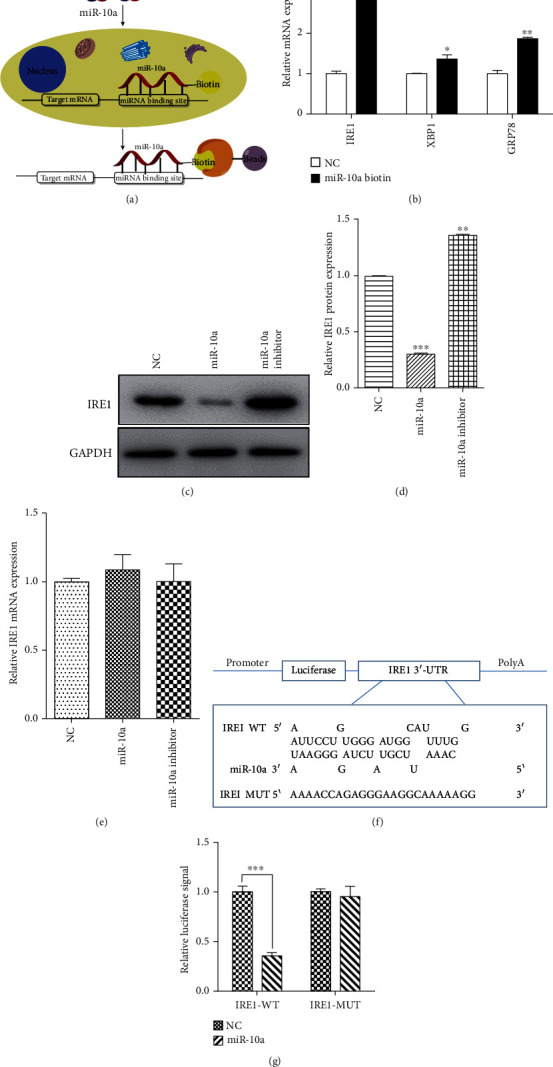
IRE1 is the target gene of miR-10a in cardiomyocytes. (a) Schematic representation of the biotinylated miRNA pull-down method. (b) The relative mRNA expression of IRE1, XBP1, and GRP78 using the biotinylated miRNA pull-down method. (c, d) The effect of the miR-10a mimic and miR-10a inhibitor on protein expression of IRE1 was detected using Western blot. (e) The effect of the miR-10a mimic and miR-10a inhibitor on mRNA expression of IRE1 was detected using qPCR. (f) Mutant sequence of IRE1 mRNA 3′UTR. (g) The effect of the miR-10a mimic on transcriptional activity of IRE1 using luciferase activity assay. Data were expressed as mean ± SD in three independent experiments. ^∗∗^*p* < 0.01 and ^∗∗∗^*p* < 0.001, compared to the NC group.

To explore the relationship between miR-10a and ERS-related proteins, we designed an affinity purification method to determine the target gene for miR-10a. Cells were transfected with biotinylated synthetic miRNAs and lysed after incubation, and miRNA and its mRNA were screened by streptavidin-coated magnetic beads (Figure 4(a)). As shown in Figure 4(b), IRE1 expression levels were higher than other ERS-related proteins including XBP1 and GRP78. We further examined the relationship between miR-10a and IRE1 by Western blot (Figure 4(c)). It was found that miR-10a significantly inhibited the protein expression of IRE1, while the miR-10a inhibitor reversed its inhibitory effect and promoted IRE1 protein expression (Figure 4(d)). Furthermore, qPCR detection of IRE1 mRNA expression revealed that IRE1 mRNA expression was not affected, suggesting that IRE1 is regulated by posttranscriptional regulation of miR-10a (Figure 4(e)). To further clarify that miR-10a directly regulates the transcriptional activity of IRE1, we constructed luciferase reporter gene plasmid of wild-type and mutant IRE1 separately, and the mutation pattern is shown in Figure 4(f). The results showed that the miR-10a mimic significantly reduced the transcriptional activity of wild-type IRE1 compared to the control group (Figure 4(g)). Combined with the above results, we confirmed that miR-10a directly inhibited the expression of IRE1.

### 3.5. Regulation of ERS by miR-10a in Cardiomyocytes

**Figure 5:**
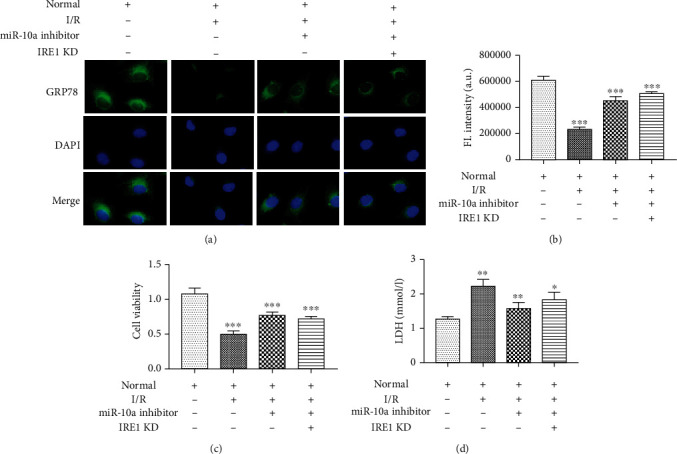
Regulation of ERS by miR-10a in cardiomyocytes. (a) The effect of the miR-10a inhibitor and knockdown IRE1 on expression of GRP78 was detected by immunofluorescence. Scale bar = 20 *μ*m. (b) Fluorescence intensity of each group in (a). (c) The effect of the miR-10a inhibitor and knockdown IRE1 on cell viability of each group was detected by MTT analysis. (d) The effect of the miR-10a inhibitor and knockdown IRE1 on LDH release of each group was detected by LDH analysis. Data were expressed as mean ± SD in three independent experiments. ^∗∗^*p* < 0.01 and ^∗∗∗^*p* < 0.001, compared to the NC group.

After confirming that miR-10a targets IRE1, we further explored its effects on ERS in cardiomyocytes. We detected the expression of the ERS marker protein GRP78 by fluorescence (Figure 5(a)). By measuring the fluorescence intensity, we found that when I/R occurs, the expression of GRP78 in cardiomyocytes is significantly inhibited, the miR-10a inhibitor will increase the expression of GRP78 on the basis of H/R injury, and the GRP78 expression increase could be inhibited when IRE1 expression is inhibited. (Figure 5(b)). The results further indicated that miR-10a inhibits moderate ERS and played the roles through IRE1 gene. In addition, we also examined cardiomyocyte viability by MTT and LDH analyses (Figures 5(c) and 5(d)). The results showed that the miR-10a inhibitor attenuated the damage of H/R on cell viability, and knockdown IRE1 can reverse myocardial cell viability.

### 3.6. Regulation of ERS by the miR-10a Inhibitor in the Rat Myocardium

**Figure 6:**
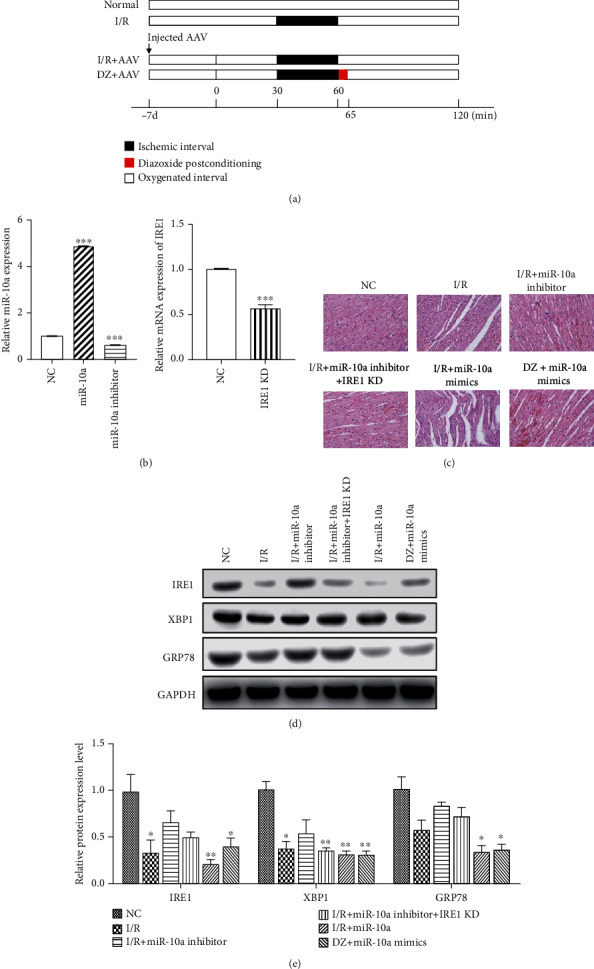
Regulation of ERS by the miR-10a inhibitor in rat myocardial tissue. (a) The schematic diagram of the established myocardial I/R injury rat model and injected AAV. (b) qRT-PCR was used to detect the miR-10a and IRE1 expression. (c) Rat myocardial tissues in different groups were detected by HE staining. Scale bar = 20 *μ*m. (d) The effects of the miR-10a inhibitor on protein expressions of GRP78, IRE1, and XBP1 were detected using Western blot. (e) Histogram summarizing results in (d). Data were expressed as mean ± SD (*n* = 6). ^∗∗^*p* < 0.01 and ^∗∗∗^*p* < 0.001, compared to the NC group.

At the cellular level, we have verified the regulation circuitry of DZ against myocardial I/R injury. We found that the miR-10a inhibitor can promote moderate ERS and reduce the effect of I/R injury on myocardial cell viability, thus protecting cardiomyocytes. We further verified this conclusion in the rat I/R model, as shown in Figure 6(a). The miR-10a and IRE1 expression change was determined by qRT-PCR (Figure 6(b)). As shown by the HE results, the miR-10a inhibitor was used on the basis of the I/R model, and the sarcomere morphology was relatively intact, and the myofilament was arranged neatly compared with the I/R group. Downregulation of IRE1 in the I/R rats attenuates the effect of the miR-10a inhibitor. miR-10a mimics impact the sarcomere morphology, and the arrangement of the myofilament is contrary to the miR-10a inhibitor. DZ also slows down the effect of miR-10a mimics in myocardial tissues (Figure 6(c)). Subsequently, we examined the expression of ERS-related proteins by Western blot (Figure 6(d)). The results indicated that the miR-10a inhibitor reverses the decrease in ERS-related protein expression caused by I/R injury, and reduced IRE1 expression could retard the effect of the miR-10a inhibitor in ERS-related protein expression. miR-10a mimics further decrease the ERS-related protein expression; DZ reverses the decrease in ERS-related protein expression by miRNA mimics transfected (Figure 6(e)). This part of the results confirmed that DZ protects myocardial tissue through miR-10a expression inhibition; the miR-10a inhibitor can reverse myocardial damage caused by I/R injury and protect myocardial tissue by promoting moderate ERS.

## 4. Discussion

Ischemic cardiomyopathy is one of the most common life-threatening diseases. Studies have shown that oxygen free radicals and intracellular calcium overload during reperfusion may be an important path of myocardial I/R injury [30–32]. Diazoxide (DZ) has been demonstrated to limit myocardial or cerebral I/R injury and apoptosis. However, the specific mechanism has not been fully elucidated. In this study, the rat model of myocardial I/R injury and myocardial H/R model were established to detect the changes of miRNA and the expression of ERS-related molecules to investigate the effect of DZ on myocardial I/R injury.

The ER is an important place for protein synthesis, folding, modification, and storage of Ca^2+^, which is of great significance for maintaining normal physiological functions. Pathological factors could induce an imbalance of ER homeostasis and dysfunction, leading to the ERS state [33]. Studies have shown that moderate ERS is a protective mechanism for cells to cope with various stresses. ERS could reduce cell damage by treating abnormal proteins, regulating calcium concentration, and restoring homeostasis. However, persistent or excessive ERS can cause cell death by a series of cascades of activation-related factors [34].

GRP78 is a marker protein of ERS and mediates the processing and maturation of nascent proteins. GRP78 is a key regulatory protein that promotes cell protein maturation, cell function, and life under normal growth conditions [35]. IRE1 is a type I transmembrane protein localized on the ER membrane with both kinase and endonuclease activities. Under normal physiological conditions, IRE1 binds to GRP78 and is inactive, but when ERS occurs, they are separated from each other and become activated [36]. The IRE1-XBP1 signaling pathway plays an important role in the ERS reaction [37, 38]. Western blot and HE results in the present studies showed that I/R could significantly downregulate the expression of IRE1 and XBP1 in the early stage the of I/R group compared with the sham operation group, indicating that I/R can inhibit the occurrence of ERS at the early stage. Diazoxide (DZ) is a highly selective mitochondrial adenosine triphosphate-sensitive potassium channel opener [39]. Its protective effect on I/R injury has been confirmed in many organs such as the heart and brain [40, 41]. Myocardial I/R injury studies have shown that DZ treatment can induce moderate ERS and reduce myocardial damage. The results of this study showed that the myocardial damage in the DZ+I/R group was significantly lower than that in the I/R group, and the ultrastructural damage of the myocardium was smaller, indicating that DZ treatment could reduce myocardial I/R injury. At the same time, among the several miRNAs treated with DZ, the change of miR-10a was the most significant, and the expression level was significantly inhibited. We found that the expression of miR-10a under I/R conditions was significantly increased, and it exerted I/R damage effects both in vitro and in vivo. In vitro studies found that when the expression of miR-10a was inhibited, the damage was reversed, which resulted in a similar effect of DZ treatment. At the same time, it was found that the miR-10a inhibitor and DZ may inhibit the release of Bcl-2, inhibit the opening of the mitochondrial permeability transition pore, and reduce the release of cytochrome in mitochondria of myocardial cells during I/R injury, thereby inhibiting cardiomyocyte apoptosis induced by I/R to play a protective role.

The effects of apoptosis and apoptosis-related gene expression preliminarily confirmed the protective effects of diazoxide and miR-10a inhibitors on cardiomyocyte apoptosis induced by H/R. Further study on the effect of miR-10a on pathomorphology and ERS of myocardial I/R injury clarified that miR-10a promoted myocardial I/R injury by downregulating ERS-related proteins IRE1, thereby inhibiting the IREl-XBP1 signaling pathway to aggravate myocardial I/R injury. This study explored the protective mechanism of DZ in myocardial I/R injury and provided a theoretical basis for clinical application of drug preconditioning to prevent myocardial I/R injury.

## Data Availability

All processed data and models used during the study are available from the corresponding authors by request. But the raw data required to reproduce these findings cannot be shared at this time as the data also forms part of an ongoing study.

## Supplementary Materials

Supplementary materials Rat primary cardiomyocytes were isolated from rat cardiac tissue and identified by ACTA1 immunofluorescence.

## References

[1] Takasu O., Gaut J. P., Watanabe E. (2013). Mechanisms of cardiac and renal dysfunction in patients dying of sepsis. *American Journal of Respiratory and Critical Care Medicine*.

[2] He S., Wang X., Chen A. (2017). Myocardial ischemia/reperfusion injury: the role of adaptor proteins Crk. *Perfusion*.

[3] Dirnagl U., Simon R. P., Hallenbeck J. M. (2003). Ischemic tolerance and endogenous neuroprotection. *Trends in Neurosciences*.

[4] Liu D., Lu C., Wan R., Auyeung W. W., Mattson M. P. (2016). Activation of mitochondrial ATP-dependent potassium channels protects neurons against ischemia-induced death by a mechanism involving suppression of Bax translocation and CytochromecRelease. *Journal of Cerebral Blood Flow and Metabolism*.

[5] Shimizu K., Lacza Z., Rajapakse N., Horiguchi T., Snipes J., Busija D. W. (2002). MitoK(ATP) opener, diazoxide, reduces neuronal damage after middle cerebral artery occlusion in the rat. *American Journal of Physiology. Heart and Circulatory Physiology*.

[6] Hausenloy D. J., Yellon D. M., Mani-Babu S., Duchen M. R. (2004). Preconditioning protects by inhibiting the mitochondrial permeability transition. *American Journal of Physiology. Heart and Circulatory Physiology*.

[7] Wu L., Shen F., Lin L., Zhang X., Bruce I. C., Xia Q. (2006). The neuroprotection conferred by activating the mitochondrial ATP-sensitive K+ channel is mediated by inhibiting the mitochondrial permeability transition pore. *Neuroscience Letters*.

[8] Busija D. W., Katakam P., Rajapakse N. C. (2005). Effects of ATP-sensitive potassium channel activators diazoxide and BMS-191095 on membrane potential and reactive oxygen species production in isolated piglet mitochondria. *Brain Research Bulletin*.

[9] He X., Mo X., Gu H. (2008). Neuroprotective effect of diazoxide on brain injury induced by cerebral ischemia/reperfusion during deep hypothermia. *Journal of the Neurological Sciences*.

[10] Wang L., Zhu Q. L., Wang G. Z. (2011). The protective roles of mitochondrial ATP-sensitive potassium channels during hypoxia-ischemia-reperfusion in brain. *Neuroscience Letters*.

[11] Tüfekci K. U., Öner M. G., Meuwissen R. L. J., Genç Ş. (2014). The role of microRNAs in human diseases. *miRNomics: MicroRNA Biology and Computational Analysis*.

[12] Bhaskaran M., Mohan M. (2013). MicroRNAs: history, biogenesis, and their evolving role in animal development and disease. *Veterinary Pathology*.

[13] Duygu B., de Windt L. J., da Costa Martins P. A. (2016). Targeting microRNAs in heart failure. *Trends in Cardiovascular Medicine*.

[14] Thum T., Galuppo P., Wolf C. (2007). MicroRNAs in the human heart: a clue to fetal gene reprogramming in heart failure. *Circulation*.

[15] Foley N. H., Bray I., Watters K. M. (2011). MicroRNAs 10a and 10b are potent inducers of neuroblastoma cell differentiation through targeting of nuclear receptor corepressor 2. *Cell Death and Differentiation*.

[16] Huang H., Xie C., Sun X., Ritchie R. P., Zhang J., Chen Y. E. (2010). miR-10a contributes to retinoid acid-induced smooth muscle cell differentiation. ***Journal of Biological Chemistry***.

[17] Yigit M. V., Ghosh S. K., Kumar M. (2017). Context-dependent differences in miR-10b breast oncogenesis can be targeted for the prevention and arrest of lymph node metastasis. *Oncogene*.

[18] Bloomston M., Frankel W. L., Petrocca F. (2007). MicroRNA expression patterns to differentiate pancreatic adenocarcinoma from normal pancreas and chronic pancreatitis. *JAMA*.

[19] Varendi K., Kumar A., Harma M. A., Andressoo J. O. (2014). miR-1, miR-10b, miR-155, and miR-191 are novel regulators of BDNF. ***Cellular and Molecular Life Sciences***.

[20] Wang X., Ling C. C., Li L. (2016). MicroRNA-10a/10b represses a novel target gene mib1 to regulate angiogenesis. *Cardiovascular Research*.

[21] Schwarz D. S., Blower M. D. (2016). The endoplasmic reticulum: structure, function and response to cellular signaling. *Cellular and Molecular Life Sciences*.

[22] Oakes S. A., Papa F. R. (2015). The role of endoplasmic reticulum stress in human pathology. *Annual Review of Pathology*.

[23] Nishikawa S., Brodsky J. L., Nakatsukasa K. (2005). Roles of molecular chaperones in endoplasmic reticulum (ER) quality control and ER-associated degradation (ERAD). *Journal of Biochemistry*.

[24] Sari F. R., Watanabe K., Widyantoro B. (2011). Sex differences play a role in cardiac endoplasmic reticulum stress (ERS) and ERS-initiated apoptosis induced by pressure overload and thapsigargin. *Cardiovascular Pathology*.

[25] Li J., Zhou W., Chen W., Wang H., Zhang Y., Yu T. (2020). Mechanism of the hypoxia inducible factor 1/hypoxic response element pathway in rat myocardial ischemia/diazoxide post‑conditioning. *Molecular Medicine Reports*.

[26] Wang D., Chen Y., Liu M. (2020). The long noncoding RNA Arrl1 inhibits neurite outgrowth by functioning as a competing endogenous RNA during neuronal regeneration in rats. *The Journal of Biological Chemistry*.

[27] Qin Y., Zheng B., Yang G. S. (2020). Tanshinone IIA inhibits VSMC inflammation and proliferation _in vivo_ and _in vitro_ by downregulating miR-712-5p expression. *European Journal of Pharmacology*.

[28] Peng X. P., Huang L., Liu Z. H. (2016). miRNA-133a attenuates lipid accumulation via TR4-CD36 pathway in macrophages. *Biochimie*.

[29] Liu Y., Liu R., Yang F. (2017). miR-19a promotes colorectal cancer proliferation and migration by targeting TIA1. *Molecular Cancer*.

[30] Kalogeris T., Baines C. P., Krenz M., Korthuis R. J. (2016). Ischemia/reperfusion. *Comprehensive Physiology*.

[31] Meng Y., Li W. Z., Shi Y. W., Zhou B. F., Ma R., Li W. P. (2016). Danshensu protects against ischemia/reperfusion injury and inhibits the apoptosis of H9c2 cells by reducing the calcium overload through the p-JNK-NF-*κ*B-TRPC6 pathway. *International Journal of Molecular Medicine*.

[32] Wang X. B., Huang X. M., Ochs T. (2011). Effect of sulfur dioxide preconditioning on rat myocardial ischemia/reperfusion injury by inducing endoplasmic reticulum stress. *Basic Research in Cardiology*.

[33] Agellon L. B., Michalak M. (2017). The endoplasmic reticulum and the cellular reticular network. *Advances in Experimental Medicine and Biology*.

[34] Yu Y., Zhang L., Liu Q., Tang L., Sun H., Guo H. (2015). Endoplasmic reticulum stress preconditioning antagonizes low-density lipoprotein-induced inflammation in human mesangial cells through upregulation of XBP1 and suppression of the IRE1*α*/IKK/NF-*κ*B pathway. *Molecular Medicine Reports*.

[35] Zhang L., Tian Z., Li W., Wang X., Man Z., Sun S. (2017). Inhibitory effect of quercetin on titanium particle induced endoplasmic reticulum stress related apoptosis and in vivo osteolysis. *Bioscience Reports*.

[36] Chen Y., Brandizzi F. (2013). IRE1: ER stress sensor and cell fate executor. *Trends in Cell Biology*.

[37] Zhang M., Han N., Jiang Y. (2018). EGFR confers radioresistance in human oropharyngeal carcinoma by activating endoplasmic reticulum stress signaling PERK-eIF2*α*-GRP94 and IRE1*α*-XBP1-GRP78. *Cancer Medicine*.

[38] Cao X., He Y., Li X., Xu Y., Liu X. (2019). The IRE1*α*-XBP1 pathway function in hypoxia-induced pulmonary vascular remodeling, is upregulated by quercetin, inhibits apoptosis and partially reverses the effect of quercetin in PASMCs. *American Journal of Translational Research*.

[39] Koch-Weser J. (1976). Diazoxide. *The New England Journal of Medicine*.

[40] Dong H., Wang S., Zhang Z., Yu A., Liu Z. (2014). The effect of mitochondrial calcium uniporter opener spermine on diazoxide against focal cerebral ischemia--reperfusion injury in rats. *Journal of Stroke and Cerebrovascular Diseases*.

[41] Lei X., Lei L., Zhang Z., Cheng Y. (2018). Diazoxide inhibits of ER stress‑mediated apoptosis during oxygen‑glucose deprivation in vitro and cerebral ischemia‑reperfusion in vivo. *Molecular Medicine Reports*.

