# Supplementary material for: Diazoxide Protects against Myocardial Ischemia/Reperfusion Injury by Moderating ERS via Regulation of the miR-10a/IRE1 Pathway
Source: Oxid Med Cell Longev. 2020 Sep 8;2020:4957238. doi: 10.1155/2020/4957238 (PMC7495230; doi:10.1155/2020/4957238)
Supplement: Supplementary materials — Rat primary cardiomyocytes were isolated from rat cardiac tissue and identified by ACTA1 immunofluorescence. [file 4957238.f1.docx]

**Supplementary data**


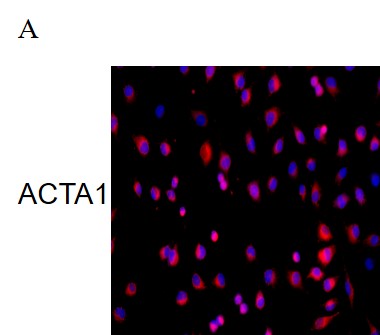


Rat primary cardiomyocytes were isolated from rat cardiac tissue and identified by ACTA1 immunofluorescence.
